# Developmental Study on “Smart Silver Care”: A Mobile Application to Alleviate Loneliness in Older Adults within the Community

**DOI:** 10.3390/healthcare11172376

**Published:** 2023-08-23

**Authors:** Hee-Kyung Choi, Kayoung Lee, Seon-Heui Lee

**Affiliations:** Department of Nursing Science, College of Nursing, Gachon University, Incheon 21936, Republic of Korea; hcgsf0910@gachon.ac.kr (H.-K.C.); kayolee@gachon.ac.kr (K.L.)

**Keywords:** aging, community, loneliness, mobile application, development

## Abstract

Background: Loneliness poses a significant threat to the quality of life of older adults. Therefore, it is essential to implement non-face-to-face services to solve the loneliness of older adults in the community. Objectives: This study used the ADDIE (Analysis, Design, Development, Implementation, and Evaluation) model to develop mobile applications as a loneliness intervention for older adults living in the community. Methods: A mobile application was developed using the ADDIE model to alleviate loneliness in older adults living in the community. The development process included a systematic review, a face-to-face preference survey, and an experts’ evaluation. From 11 to 15 June 2021, the following six databases were used to search for related articles: Ovid-Medline, Ovid-EMBASE, Cochrane Library, KISS, Korea Med, RISS. A preference analysis was conducted on 100 adults aged 65 or older living in the community from 15 July to 31 August 2021. Results: A mobile application for community-dwelling older adults was developed. Its contents included emotional support, cognition, physical activity, health data, nutrition, and motivation. They were organized through a systematic review and preference survey in the analysis stage. They were also designed as main menus and sub-content at the design stage. They also designed the structure, functionality, and interface layout. The application was developed by integrating the designed content and determining the operating system, language, access method, privacy, and server history. Then, experts evaluated the validity of the application. Conclusions: The prototype mobile application incorporates emotional support, cognition, physical activity, health data, nutrition, and motivation. It is expected to help older adults achieve their goals by promoting participation. By incorporating expert validity into the content development process of early prototypes, we have improved the usability and acceptability of our products. Future pilot trials are needed to evaluate the effectiveness of this mobile application among older adults.

## 1. Introduction

### 1.1. Background

The proportion of older adults is expected to increase significantly based on current population trends [1]. Loneliness in older adults increases because of the deterioration of their physical function and the decrease in their social roles and networks [2]. It threatens their quality of life and increases the risk of dementia, heart diseases, and mortality [3,4,5,6]. Therefore, it is essential to develop interventions to alleviate loneliness [7].

Although local governments have provided face-to-face services to older adults, including medical and nursing care, their loneliness has not been reduced. The scarcity of human resources and limited access to services pose challenges in utilizing resources efficiently and maintaining consistent operations [8]. Kim [9] reported that the quality of care services can be improved using information and communication technology (ICT). Non-face-to-face services are emerging as a means to improve older adults’ social participation and health status by providing loneliness interventions [10,11,12].

Therefore, non-face-to-face services must be developed to reduce loneliness among older adults. Previous studies have reported the development of non-face-to-face services, including emotional support, physical activity, cognitive activity, and nutritional education, to reduce loneliness among older adults [13,14,15,16]. These non-face-to-face services were developed considering older people’s preferences and overcoming technical barriers. Specifically, such services have been reported to reduce loneliness through emotional support, such as communicating with others online and exercising together [17,18]. It positively impacts emotions through non-face-to-face services, such as cognitive activities and nutrition education [13,19].

Previous systematic reviews have investigated the effectiveness of non-face-to-face services as loneliness interventions for community-dwelling older adults. Additionally, studies have examined attitudes and intentions [20,21] and confirmed the perception, preference, and acceptance of ICT among older adults [22,23]. However, studies on developing evidence-based mobile applications to reduce loneliness among older adults are limited.

### 1.2. Objectives

Therefore, this study aimed to develop an evidence-based mobile application to reduce loneliness among older adults. The mobile application incorporates educational interventions to improve the health of older adults and thereby provides various forms of emotional support to alleviate loneliness. To develop the application, we employed the analysis, design, development, implementation, and evaluation (ADDIE) model, a recognized educational design method. Furthermore, previous studies on developing non-face-to-face services for older people adopted the ADDIE model as the development methodology [24,25,26,27,28,29,30].

## 2. Methods

### 2.1. Ethics Statement

The Institutional Review Board (IRB) of Gachon University (IRB No. 1044396-202102-HR-019-02) approved this study.

### 2.2. Development Process

**Figure 1:**
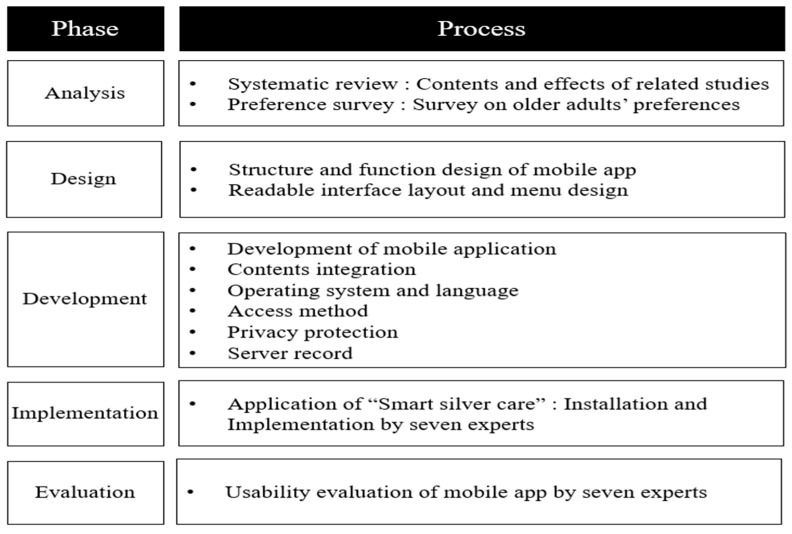
Development phase and process of the mobile application using analysis, design, development, implementation, and evaluation (ADDIE) model.

This study developed a mobile application to reduce loneliness among community-dwelling older adults. We followed the ADDIE model (Figure 1), which includes an ongoing review of objectives, interrelationships between elements, and modifications based on real-world experiences [27]. The model uses five development phases: (a) analysis, (b) design, (c) development, (d) implementation, and (e) evaluation. It describes the process of creating a mobile application to reduce loneliness among older people in a community [24,25,26].

#### 2.2.1. Phase 1: Analysis

The analysis phase includes specific research techniques, such as a literature review and needs analysis, to define problems and determine possible solutions [31]. In this study, the content of mobile device applications was organized through a systematic review and preference survey to develop evidence-based mobile device applications.

First, a systematic review was conducted to identify non-face-to-face service types and components and understand the effects of each type. Relevant articles were searched using the following databases: Ovid-MEDLINE, Ovid-EMBASE, Cochrane Library, RISS, KISS, and KoreaMed. We searched for articles using keywords and medical subject headings (MeSH). The search was conducted using the appropriate Boolean operators “AND” or “OR”. The inclusion criteria were (a) older adults, (b) non-face-to-face services developed for older adults, and (c) loneliness among older adults. We excluded studies not written in Korean or English, duplicate studies, animal studies, abstracts, conference posters, and review articles. Two authors independently selected the studies based on the predefined inclusion and exclusion criteria and agreed to select the appropriate studies.

Based on our systematic review and the literature [32,33], we developed a questionnaire to investigate older adults’ preferences for mobile applications. The questionnaire included (a) the general characteristics of the participants and (b) the preferred components of non-face-to-face services. A five-point Likert scale with scores ranging from 1 (not preferred at all) to 5 (highly preferred) was used to rate the component categories. The Likert scale offered a range of categories for participants to choose from [34]. Additionally, a change in the Likert scale was easier to interpret when the patient’s level changed from one category to another [35]. Therefore, clinically significant changes might be easier to identify using a Likert scale. The item-level content validity index (I-CVI) was calculated by three experts (professors majoring in nursing for older adults).

We conducted a face-to-face survey to confirm the preference for non-face-to-face services among 100 community-dwelling older adults from 15 July to 31 August 2021. An older person is one whose physical, psychological, and social functions decline as they experience aging in the last stage of their life cycle [36]. The criteria for including participants in the questionnaire were (a) adults aged 65 years or above and (b) living in local communities. The exclusion criteria were (a) cognitive or (b) communication impairment. The frequency, percentage, average, and standard deviation (SD) of all data were used to analyze the general characteristics of participants and preferences for non-face-to-face services. This study employed SPSS Windows software version 26.0.

#### 2.2.2. Phase 2: Design and Phase 3: Development

During the analysis phase, mobile application content was designed explicitly based on the findings and insights obtained—the main content comprised emotional support, cognition, physical activity, health data, and nutrition. The structure and function of the mobile application were designed during the design phase. Moreover, the interface layout, main menu, and sub-contents were designed to increase readability and ease of use. The mobile application content was developed with company programmers during the development phase. Decisions were made regarding the operating system, programming language, access methods, downloads and installations, privacy protection, and server records.

#### 2.2.3. Phase 4: Implementation and Phase 5: Evaluation

For the developed mobile application, seven experts used and evaluated the mobile application’s content for community-dwelling older adults at this phase. The experts included three nursing professors majoring in nursing for older adults, three gerontological nurse practitioners, and one administrator in charge of long-term care insurance for older adults at the National Health Insurance Service. After developing the content, the I-CVI was calculated by seven experts using a four-point Likert scale regarding the validity of the developed application for older adults in the community. Content validity is a process in which experts subjectively determine whether the content includes all the essential elements. In a previous study, three to 10 experts were considered adequate to calculate CVI, and I-CVI scores higher than 0.8 were considered reasonable [37]. Therefore, in this study, 7 experts calculated CVI by evaluating the application.

## 3. Results

### 3.1. Analysis Phase Findings: A Systematic Review

A total of 15 studies were selected through a systematic review. Through a systematic review, we identified five domains of non-face-to-face care services: emotional support, cognition, physical activity, health data, and nutritional management (Table 1). Of the 15 studies selected, nine examined the effects of ICT-based non-face-to-face services on loneliness and social isolation, and seven focused on the outcome of social support and quality of life. Non-face-to-face services positively affect loneliness, social isolation, social support, and quality of life [33].

First, “emotional support” was evaluated using an artificial intelligence (AI) speaker, video calls, and an online community in 14 studies. The AI speaker was used in eight studies, where the device expressed emotions, responded to stimuli, asked questions, or provided feedback on activities. The studies had varying durations, ranging from one week to 8 weeks, and were conducted with different frequencies, ranging from once a week to once every seven weeks. The studies involved conversations ranging from two minutes to 10 min per session or from 20 min to 10 h a day. Video calls were conducted in three studies, allowing free interaction with family or friends or on specified topics. The duration of these studies ranged from three to six months, with one study having at least five minutes a week. Three studies focused on an online community where participants shared photos or videos, sent messages, and wrote diaries. These studies ranged from eight weeks to three months, without a specific frequency or time mentioned. Two studies were conducted, involving both group discussions and individual sessions. One study comprised real-time lectures conducted five times a week, with sessions lasting between 30 min and one hour. The specific duration of the study was not mentioned.

Second, in three studies, “cognition” was targeted through schedule management and a cognitive game. The cognitive game involved a word game designed to enhance cognitive functions and enjoyment for older adults. The duration of this study ranged from eight weeks to three months, without specific mention of frequency and time. Additionally, three studies focused on schedule management, where participants utilized a calendar and entered their schedules to receive notifications.

Third, in two studies, “physical activity” was incorporated through games and music exercises. The exercises included upper- and lower-extremity movements and stretching exercises designed as enjoyable virtual reality games, considering the physical ability of older adults. It was conducted twice a week for six weeks, with each episode lasting 50 min. The first episode, which involved exercise with music, was conducted five times a week in real-time, with each episode lasting between 30 min and one hour.

Fourth, in three studies, “health data” were conducted, including step count and vital signs. Daily step counts were measured, and feedback was given in the following days, with durations ranging from one week to 8 weeks. In one study, vital signs such as blood pressure, pulse, and oxygen saturation were measured, but no specific details regarding the period, frequency, and time were mentioned.

Fifth, only one study included “nutritional management” through nutrition education, daily records and feedback on dietary habits, and a recommended diet intake amount. The intervention lasted eight weeks, but frequency or time details were not mentioned.

### 3.2. Analysis Phase Findings: A Preference Analysis

The questionnaire was administered to 100 community-dwelling adults aged 65 years and above, in a preference survey. The survey was conducted on a five-point Likert scale. The scale comprised scores ranging from 1 (not preferred at all) to 5 points (highly preferred), with 3 points representing “neutral”. Three experts reviewed the questionnaire and calculated the average CVI score as 0.99. The questionnaire was developed by requesting additional measures to motivate users. The content validity survey responded to the validity question with “4 points for very valid”, “3 for valid”, “2 for not valid”, and “1 for very invalid”. The developed questionnaire included and analyzed (a) the general characteristics of the participants and (b) their preferred components of the non-face-to-face services. Statistical analysis was conducted using SPSS for frequency, percentage, mean, and SD. Table 2 shows the results.

The mean age of older adults was 73.2 ± 7.0 years, of which 67% were women. Each item was evaluated on a five-point Likert scale—the higher the score, the better the preference (Table 2). The preferences for the mobile application’s contents, such as the “emotional support” (3.22 ± 0.23), “cognition” (3.09 ± 0.22), and “health data” (3.47 ± 0.28) categories, were positive at more than three points. However, preference was relatively low for the “physical activity” (2.99 ± 1.28), “nutritional management” (2.94 ± 0.23), and “motivation” (2.86 ± 1.20) categories. In additional questions, participants indicated that “twice a week” was appropriate for the number of interventions and that they preferred “less than 30 min” per episode.

### 3.3. Findings of the Design Phase

**Figure 2:**
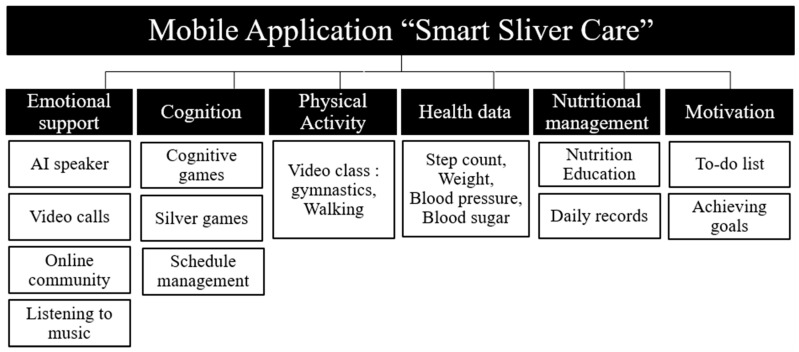
Diagram of the main menu and sub-content of the mobile application “Smart Silver Care”.

The structure and function of the mobile application were established, and the main menu was designed (Figure 2). The main menu content comprised six types, emotional support, cognition, physical activity, health data, nutrition management, and motivation, and each type included sub-content (Table 3). The interface layout was also placed, and readability was improved so that older adults could easily access the menu. Each menu was designed to make it easy for older people to use the application by adequately utilizing messages, images, videos, and voices.

Emotional support content consists of “AI speaker”, “video calls”, “online community”, and “listening to music”. First, older adults use voice recognition using the “AI speaker” function to talk to the AI in a mobile application. Second, users can select a family member, friend, or administrator on the mobile application and connect with them through “video calls”. Third, using an “online community”, users can upload photos to a community hub and communicate with other users. Fourth, the application connects to preferred music for “listening to music”, thereby enabling users to listen to music such as trots, hymns, and pop songs.

The cognition content consists of “cognitive games”, “silver games”, and “schedule management”. First, “cognitive games” train users to encourage concentration, memory, language and thinking, and spatiotemporal and calculation abilities. Second, in “silver games”, the older adults play the preferred games by connecting them to free apps. Third, “schedule management” records user schedules, including medication time, treatment dates, and annual meetings.

The physical activity consists of “gymnastics” and “walking” on a video taking place in real-time. First, “gymnastics” connects to participants in real-time video and trains flexibility, equilibrium, muscle strength, and body coordination to prevent dementia. Second, “walking” teaches the proper form of walking and provides healthcare tips. The walking challenge measures the number of steps required.

Health data consist of “step count”, and “weight, blood pressure, and blood sugar”, the results of which are displayed. If the measurement is abnormal, the administrator reviews it and contacts the user, if necessary.

Nutritional management consists of “nutrition education” and “daily records”. First, “nutrition education” includes healthy eating habits and videos on dietary recipes. These were developed specifically for older adults in the community and utilize larger fonts and voice prompts to make them easier to understand. Second, in “daily records”, users enter daily meat, water, vegetables, and fruit intake.

Motivation consists of a “to-do list” and “achieving goals”. Users check daily lists to determine whether they have achieved their goals. This function increases user participation and enables administrators to manage users’ current status better.

### 3.4. Findings of the Development Phase

**Figure 3:**
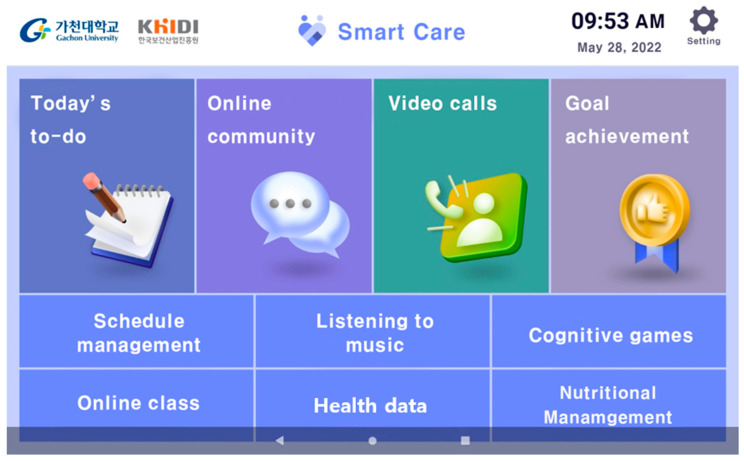

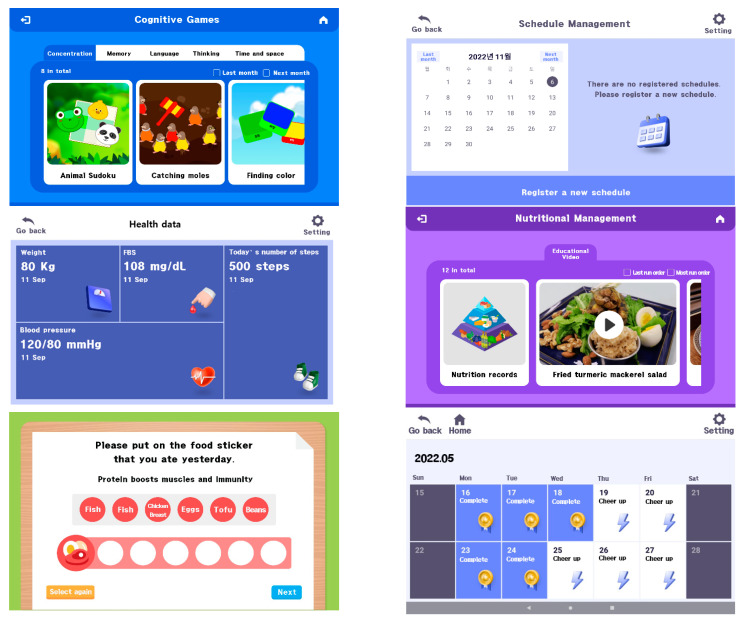
The developed mobile application “Smart Silver Care”.

This “smart silver care” mobile application was built based on the results of the analysis and design phases and developed through repeated improvements (Figure 3). We developed an application that can be used on a tablet using the Android system, and the language used is Korean. After downloading and installing, it can only be used by users registered in advance with an administrator. The administrator monitors the server and continuously resolves errors. Logs and access times remain on the server that the administrator can only view. Collecting only the content agreed upon by the user in advance protected the participant’s privacy and improved security.

### 3.5. Findings at the Implementation and Evaluation Phase: Content Validity Experts

The seven experts were three nursing professors majoring in nursing for older adults, three gerontological nurse practitioners, and one administrator in charge of long-term care insurance for older adults at the National Health Insurance Service. Given that the app was developed based on a systematic review and the survey results on the preference for non-face-to-face services of older adults in the community, it already included their opinions; therefore, it was only evaluated by experts related to older people. Experts evaluated the content validity of the developed application using a four-point Likert scale. The average I-CVI was calculated as 1.00, indicating that all contents were valid. In the experts’ opinions, items that needed to be corrected were mentioned. First, the time required for viewing each video was shortened. In response to the evaluation, it was summarized within 30 min or less. Specifically, extended configurations might be burdensome for older adults and thereby render concentrating challenging. Second, as the content was extensive, a need for prior education was raised for easy older-adult utilization. Accordingly, the participants were reminded of their goals daily on the screen, and weekly feedback and consultations were conducted with the administrator once a week. Third, in response to feedback, the content names were modified to be more engaging and easily understandable.

## 4. Discussion

This study utilized the ADDIE model to develop a mobile application to reduce loneliness among older adults in the community. The primary objective was to create an evidence-based mobile application that effectively addresses and alleviates loneliness among community-dwelling older adults. A systematic review identified five domains and specific components of mobile applications that could decrease loneliness and social isolation, and facilitate social support and quality of life [33]. Therefore, our mobile application prototype is expected to effectively address loneliness among older adults in the community by replacing face-to-face services that reflect the current situation.

This study indicated that older adults preferred mobile application content to address emotional support, cognition, and health issues. However, the preferences for physical activity, nutritional management, and motivation were relatively low. Therefore, it was necessary to develop mobile applications that considered these preferences. In previous studies, programs reflecting older adults’ preferred genres of music and cognitive games were used to confirm the emotional effects of their preferred content [48,49]. Furthermore, Ahn et al. [50] and Li, Xu, Pham, Theng, Katajapuu, and Luimula [18] reported that older adults preferred to play games for exercise, which improved their enjoyment. Previous studies reported that older adults have a positive experience when non-face-to-face services are explicitly designed for them [13,51,52,53]. Therefore, considering the preferences of the surveyed older adults while creating services and programs could stimulate their interest and motivate them to participate.

Han and Park [20] confirmed older adults’ positive attitudes and intentions to use non-face-to-face services. In addition to the results showing older adults’ growing positive perceptions of non-face-to-face services [54], our systematic review and preference survey results are consistent with these previous studies. In addition, as the mobile application was developed to motivate users and allow administrators to monitor them, user participation was expected to be high. Mobile applications can provide interventions that facilitate their use through continuous monitoring of older adults and contact with administrators [55]. Therefore, we expected the community’s older adults to receive the developed mobile application well.

A previous study reported that the interface design of a mobile application should consider the cognitive decline, physical ability, and motivational barriers of older adults. Older adults had difficulty understanding the application’s structure, including the text, buttons, and icons [56]. Similarly, when designing the structure of a mobile device application, user-friendly factors such as readability, operability, comprehension, convenience, and aesthetics must be considered to increase its usability [57]. In our study, seven experts confirmed the content validity using CVI. The CVI of the mobile application was rated positively, with a score of 3 or higher. The content was adequately considered through a systematic review and survey of preferences. Therefore, evidence-based content was appropriate and was expected to be effective when applied to older adults.

It is necessary to expand the infrastructure by mobilizing knowledge to strengthen the ability to utilize research results [58]. We are considering publishing research reports and papers on the mobile applications developed in this study, by promoting them using media, such as news and advertising, and utilizing commercial companies and products [59,60]. Additionally, the effect of the initial prototype content of the mobile application developed in this study on loneliness in older adults in the community should be verified by comparing experimental and non-experimental groups in future studies. Furthermore, finding ways to commercialize mobile applications equipped with services using AI in the future is necessary.

This study had several limitations. First, each non-face-to-face service’s components and outcome indicators varied in the systematic review. Further, the lack of experimental studies dealing with non-face-to-face services made the analysis difficult. Therefore, it is necessary to reconfirm the effects of interventions in the future using a meta-analysis of the most recent studies. Second, it is challenging to represent the entire older-adult population because of the limited number of participants in the survey. Thus, it is necessary to analyze a more significant number of participants. Third, the evaluation of the app did not go through a testing phase with the target population—older adults—which may leave doubts about its suitability for the target population. However, it is important to underline that the app has been developed in a way that reflects the preferences and opinions of older adults, collected in the analysis phase.

## 5. Conclusions

We aimed to create a mobile application for older people living in the community using the ADDIE model as the guiding framework. We provided detailed information on the developed mobile application, which is expected to reduce the loneliness of older adults in the community innovatively. The contents included emotional support, cognition, physical activity, health data, nutritional management, and motivation. The most beneficial services for older adults are those that provide them with social relationships and the ability to maintain their health and live at home [61]. Mobile applications can satisfy the needs of older adults, improve their quality of life [62], and should include services that reduce loneliness and facilitate contact with their administrators [55]. The time and frequency per session were determined by a systematic review, a survey of the older adults, and expert advice, so it is considered appropriate for use in older adults. Future research is expected to improve the time and frequency per episode, so that it does not cause delusions and hallucinations when used by older adults with cognitive impairments. The evidence-based mobile app developed in this study is expected to reduce loneliness among older adults in the community. We found it essential to evaluate usability through expert validity throughout the content development process for early prototypes. In future studies, the effect of the mobile application developed in this study on loneliness in older adults in the community should be verified by comparing the experimental and control groups.

## Figures and Tables

**Table 1:** Types and components of non-face-to-face interventions to reduce loneliness in older adults.

Type of Intervention	Author and Year	Components of Intervention	Duration, Frequency, Time
Emotional support	Cohen-Mansfield J 2021 [38]	Lecture	5 days/1 week, 90 min/1 day
	Ali R 2020 [17]	Communication with the mobile device Feedback on facial expressions	4-6 weeks, 2 days/ 1 week, 2–3 min
	Tsai HH 2020 [39]	Videotelephony	6 months, 1 day/1 week, 5 min
	Neves BB 2019 [40]	Videotelephony Community	3 months
	Jarvis MA 2019 [41]	Conversation with the mobile device Feedback on loneliness reaction	8 weeks
	Czaja SJ 2018 [14]	Community Lecture	3 months
	Koceski S 2016 [42]	Videotelephony	Not detailed
	Lara J 2016 [19]	Community	8 weeks
	Ring L 2013 [43]	Communication with the mobile device	1 week, 5–10 min
	Bank MR 2008 [44]	Communication with the mobile device	8 weeks, 1 day/1 week, 30 min/1 day
	Wada K 2007 [45]	Communication with the mobile device	3 weeks, 7 days/1 week, 10 h/1 day
	Bickmore TW 2005 [10]	Communication with the mobile device	8 weeks, 5–10 min
	Wada K 2004 [46]	Communication with the mobile device	5 weeks, 3 days/1 week, 20 min/1 day
	Kanamori M 2003 [47]	Communication with the mobile device	7 weeks, 4 days/1 week, 1 h/1 day
Physical activity	Cohen-Mansfield J 2021 [38]	Exercise with music	5 days/1 week, 90 min/1 day
	Li J 2017 [18]	Exercise game	6 weeks, 2 days/1 week, 50 min/1 day
Cognition	Czaja SJ 2018 [14]	Calendar, Game	3 months
	Koceski S 2016 [42]	Calendar, Alarm	Not detailed
	Lara J 2016 [19]	Schedule	8 weeks
Health data	Koceski S 2016 [42]	Vital sign	Not detailed
	Lara J 2016 [19]	Measurement of daily steps	8 weeks
	Ring L 2013 [43]	Measurement of daily steps	1 week, 5–10 min
	Bickmore TW 2005 [10]	Measurement of daily steps	8 weeks, 5–10 min
Nutrition	Lara J 2016 [19]	Intake input Dietary feedback	8 weeks

**Table 2:** Preference for mobile application contents of older adults in the community (N = 100).

Category	Contents		N (%)	M ± SD	Category	Contents		N (%)	M ± SD
Emotional support				3.22 ± 0.23	Physical Activity				2.99 ± 1.28
	AI speaker			2.91 ± 1.22		Exercise			2.99 ± 1.28
	Video calls			3.20 ± 1.19		Multiple responses (N = 155)	Biking	14 (9.03)	
	Online community			3.41 ± 1.25			Climbing	16 (10.32)	
	Listening to music			3.37 ± 1.15			Walking	70 (45.16)	
	Multiple responses (N = 124)	Trot	51 (41.13)				Gymnastics	8 (5.16)	
		Popular song	26 (20.97)				Running	0 (0.00)	
		Classic	9 (7.26)				Terra band	3 (1.94)	
		Traditional music	5 (4.03)				Golf	10 (6.45)	
		Hymn	31 (25.00)				Ping-pong	7 (4.52)	
		Others	1 (0.81)				Badminton	4 (2.58)	
		None	1 (0.81)				Dance	12 (7.74)	
Cognition				3.09 ± 0.22			Others	7 (4.52)	
							None	4 (2.58)	
	Cognitive games			3.33 ± 1.11	Health data				3.47 ± 0.28
	Silver games			2.90 ± 1.33		Weight, Blood pressure, Blood sugar, etc.			3.67 ± 1.17
	Multiple responses (N = 109)	Shogi	10 (9.17)			Step count			3.27 ± 1.15
		Hwatu	24 (22.02)		Nutritional management				2.94 ± 0.23
		Omok	4 (3.67)			Nutrition Education			3.10 ± 1.31
		Jigsaw puzzle	10 (9.17)			Daily records			2.78 ± 1.13
		Pairing	3 (2.75)		Motivation	To-do list, Achieving goals			2.86 ± 1.20
		Playing cards	1 (0.92)						
		Memory quiz	8 (7.34)						
		Baduk	10 (9.17)						
		Smart game	8 (7.34)						
		Others	10 (9.17)						
		None	21 (19.27)						
	Schedule management			3.05 ± 1.14					

For example, when 100 people who responded to the survey chose multiple preferred types of exercise, 14 out of 155 responses preferred to ride a bicycle, showing a 9.03 percent rate.

**Table 3:** Specific contents of the mobile application in five domains.

Domain	Contents	Details	Time	Frequency
Emotional support	AI speaker	- Search for knowledge and living information using AI voice engine functions, dictionary and translation, and interesting stories - Using TV channels	Regular use	
	Video calls	- Select family, acquaintances, and administrators and connect to a video call - Monitoring the usage history of the manager - Used for real-time feedback from administrators	More than 5 min	Twice a week
	Online community	- Take a picture of the process of planting and growing seeds and upload it to the community - Leave a comment after looking at another person’s flower garden picture - Monitoring the usage history of the manager	Twice a week	
	Listening to music	- Connect to a free app and play songs when selecting trot, hymn, pop song, etc. - Encourage and give feedback via video call	Twice a week/ Once a week (Feedback)	
Cognition	Cognitive games	- Connect to a free app and play games when selecting Hwatu, Janggi, Omok, etc. - Encourage and give feedback via video call	Twice a week/ Once a week (Feedback)	
	Silver games	- The developed data are divided into concentration, memory, language thinking ability, space–time ability, and computational ability - Monitoring the usage history of the manager	Three times a week	
	Schedule management	- View the monthly calendar to register meals, medication, medical care days, and other (anniversaries, gatherings, etc.) schedules - Monitoring the usage history of the manager	Regular use	
Physical activity	Gymnastics	- Live video to perform gymnastics with music - Flexibility, equilibrium, muscle training education for fall prevention, and physical coordination exercise education for dementia prevention - Monitoring the usage history of the manager	30 min	Twice a week
	Walking	- Training on proper walking and health care tips and operating a real-time walking challenge by measuring the number of steps	30 min	Twice a week
Health data	Step count	- Measure using the step measurement app - Recommended to walk more than 4000 steps	Regular use	
	Weight	- Data on weight are stored in the app when the user makes independent measurements using a scale.	Once a week before breakfast	
	Blood pressure	- Data on blood pressure are stored in the app when the user makes independent measurements using a blood pressure meter. - Present measurement results according to criteria - Patient and administrator notifications in case of abnormal numbers	Once a day	
	Blood sugar	- Data on blood sugar are stored in the app when the user makes independent measurements using a blood glucose meter. - Present measurement results according to criteria - Patient and administrator notifications in case of abnormal numbers	Three times a week (Diabetic) or once a month (normal) before breakfast	
Nutritional management	Nutrition education	- Select and play the produced diet training and diet recipe video - Monitoring the usage history of the manager	60 min	Once a week
	Daily records	- Record daily intake of protein, vegetables/fruit, and water, including meat - Monitoring the usage history of the manager	Five times a week	

## Data Availability

The data that support the findings of this study are available from the corresponding author, upon reasonable request.
